# Yoga Program for Type 2 Diabetes Prevention (YOGA-DP) Among High-Risk People: Qualitative Study to Explore Reasons for Non-participation in a Feasibility Randomized Controlled Trial in India

**DOI:** 10.3389/fpubh.2021.682203

**Published:** 2021-09-03

**Authors:** Pallavi Mishra, Sheila Margaret Greenfield, Tess Harris, Mark Hamer, Sarah Anne Lewis, Kavita Singh, Rukamani Nair, Somnath Mukherjee, Nandi Krishnamurthy Manjunath, David Ross Harper, Nikhil Tandon, Sanjay Kinra, Dorairaj Prabhakaran, Kaushik Chattopadhyay

**Affiliations:** ^1^Centre for Chronic Disease Control, New Delhi, India; ^2^Institute of Applied Health Research, University of Birmingham, Birmingham, United Kingdom; ^3^Population Health Research Institute, St. George's University of London, London, United Kingdom; ^4^Institute Sport Exercise and Health, Division of Surgery and Interventional Science, University College London, London, United Kingdom; ^5^Division of Epidemiology and Public Health, University of Nottingham, Nottingham, United Kingdom; ^6^Bapu Nature Cure Hospital and Yogashram, New Delhi, India; ^7^Swami Vivekananda Yoga Anusandhana Samsthana, Bengaluru, India; ^8^Harper Public Health Consulting Limited, London, United Kingdom; ^9^All India Institute of Medical Sciences, New Delhi, India; ^10^London School of Hygiene and Tropical Medicine, London, United Kingdom

**Keywords:** yoga, lifestyle, prediabetes, qualitative research, prevention, physical activity, diet, non-participation

## Abstract

**Background:** Yoga-based interventions can be effective in preventing type 2 diabetes mellitus (T2DM). We developed a Yoga program for T2DM prevention (YOGA-DP) among high-risk people and conducted a feasibility randomized controlled trial (RCT) in India. The objective of this study was to identify and explore why potential participants declined to participate in the feasibility RCT.

**Methods:** An exploratory qualitative study, using semi-structured interviews, was conducted at a Yoga center in New Delhi, India. Fourteen people (10 women and four men) who declined to participate in the feasibility RCT were interviewed, and 13 of them completed the non-participant questionnaire, which captured their socio-demographics, diets, physical activities, and reasons for declining.

**Results:** Three types of barriers were identified and explored which prevented participation in the feasibility RCT: (1) personal barriers, such as lack of time, perceived sufficiency of knowledge, preferences about self-management of health, and trust in other traditional and alternative therapies; (2) contextual barriers, such as social influences and lack of awareness about preventive care; and (3) study-related barriers, such as lack of study information, poor accessibility to the Yoga site, and lack of trust in the study methods and intervention.

**Conclusions:** We identified and explored personal, contextual, and study-related barriers to participation in a feasibility RCT in India. The findings will help to address recruitment challenges in future Yoga and other RCTs.

**Clinical Trial Registration:**www.ClinicalTrials.gov, identifier: CTRI/2019/05/018893.

## Introduction

In 2019, India had 77 million people with diabetes, and which is expected to increase to 101 million by 2030 (1). The country has more than 77 million people at high-risk of type 2 diabetes mellitus (T2DM) due to their blood glucose levels being raised, but below the established threshold for T2DM itself (2). These people are more likely to develop T2DM in the future (3). Unhealthy lifestyle factors (i.e., physical inactivity and unhealthy diet) are the major risk factors for T2DM (3). Previous studies have reported low physical activity levels and high intake of an unhealthy diet among Indians (2, 4, 5). Screening and providing an effective lifestyle intervention is a cost-effective strategy for improving blood glucose levels and preventing T2DM among high-risk individuals (3).

People's socio-cultural expectations and health beliefs need to be taken into consideration when designing and implementing health interventions (6). Systematic reviews have highlighted the beneficial effects of Yoga in T2DM and metabolic syndrome (7–10), and thus, Yoga-based interventions can play a vital role in the prevention of T2DM among high-risk individuals. These interventions may be more acceptable among Indians because Yoga has been a part of their culture and resonates with their health beliefs (11, 12). It helps discipline the body and mind through promoting physical activity and a healthy diet (13). It includes low- and moderate-intensity activities and also helps in strengthening muscles (11, 14). Yoga-based interventions are easy to replicate in a range of populations and settings. It uses a gentle approach and requires a low to moderate level of guidance to learn (11). It is inexpensive and can be practiced indoors and outdoors (11). It is usually safe, even in individuals with a wide range of comorbidities (11, 14).

We developed a Yoga program for T2DM prevention (YOGA-DP) among high-risk people in India. YOGA-DP is a structured lifestyle education and exercise program, provided over a period of 24 weeks, and the exercise part is based on Yoga (15). We plan to conduct a randomized controlled trial (RCT), which will assess whether YOGA-DP is effective in preventing T2DM among high-risk people in India. We conducted a feasibility RCT to determine the feasibility of undertaking the main RCT (16). As part of this work, we carried out semi-structured interviews to identify and explore why potential participants declined to participate. The findings will help us in designing and successfully conducting the main RCT.

## Methods

An exploratory qualitative study was conducted as part of the feasibility RCT. The detailed study protocol is published elsewhere (16). Briefly, the feasibility RCT was conducted at two Yoga centers in India—one in northern India [Bapu Nature Cure Hospital and Yogashram (BNCHY, New Delhi)] and one in southern India [Swami Vivekananda Yoga Anusandhana Samsthana (SVYASA, Bengaluru)]. A multipronged approach, such as advertisements, door-to-door visits, and screening camps, was used to identify potential participants, i.e., adults at high-risk of T2DM. Those with fasting blood glucose levels of 5.6–6.9 mmol/L (i.e., 100–125 mg/dL) were considered at high-risk of T2DM (17).

In total, 727 individuals were approached to participate in the study. These individuals were given an information sheet prior to the capillary blood test. The information sheet contained detailed information about the purpose of the study, why an individual was approached to participate, what this study would involve including study-related processes (blood tests and the YOGA-DP program), recruitment and randomization to the two study arms, possible disadvantages and benefits of participation, and confidentiality of the data. Of those, 16 (11 at BNCHY and five at SVYASA) were either found to be overaged or diabetic. The remaining 711 (406 at BNCHY and 305 at SVYASA) people were invited for screening by capillary blood test using a glucometer. After the glucometer test, 551 (331 at BNCHY and 220 at SVYASA) individuals were excluded because of screen failure, non-participation by the potential participants, and the incorrect result given by a batch of glucometer strips. At BNCHY, 61 (15%) potential participants declined to come for the confirmatory venous blood test after the glucometer test, which was to ensure their eligibility for recruitment in the study. At SVYASA, all the 85 potential participants found eligible after the glucometer test came for the venous blood test, and none withdrew from the study. A reason for this difference in uptake between the sites may be that most of the participants recruited in the study at SVYASA were living close to SVYASA, which is on the outskirts of the city. Some of the study participants also reiterated the same reason for their participation in the study during interviews at the end of the study. The staff at BNCHY contacted all the 61 potential participants who declined to come for the confirmatory venous blood test and invited them to fill in a non-participant questionnaire and take part in a semi-structured interview with a trained qualitative researcher from the Centre for Chronic Disease Control (CCDC), India.

Therefore, in this qualitative study, participants were only recruited at BNCHY. All those who declined to participate in the feasibility RCT were requested to take part in a semi-structured interview to identify and explore their reasons for non-participation. Fourteen agreed, and 13 of these also provided socio-demographic details, information on diet and physical activity, and reasons for non-participation at the beginning of the interview. Although we started reaching data saturation (18, 19) by the seventh and eighth interview, we conducted interviews with all the 14 participants to ensure that we did not miss any new and unique information and to provide a broader socio-demographic spread of these participants.

The trained qualitative researcher conducted the semi-structured interviews either face-to-face or by telephone from August 2019 to March 2020 by prior appointment. Face-to-face interviews were conducted at the participant's house or BNCHY. This researcher was not involved in the recruitment of participants in the feasibility RCT. The interviews were conducted using a pre-tested interview guide in Hindi, a local language, as preferred by the participants. The interview guide had some key open-ended questions which were adapted to the participants' responses to capture detail and develop a deeper understanding of each answer. The topics covered were: personal and health profiles of the participants, their diets and physical activities, knowledge about T2DM and Yoga, information and perceptions about the YOGA-DP feasibility RCT, and reasons for non-participation in the study. The interviews were digitally recorded, transcribed verbatim by a professional transcriber, and then translated from Hindi to English by a professional translator (19). The quality of the transcripts was ensured by the researcher through listening to the recordings and constantly comparing them to transcripts to rule out the possibility of missing data.

During and after data collection, the qualitative researcher familiarized herself with the data by reading the transcripts multiple times. The researcher coded all the transcripts using the interpretative approach and deductive logic with the help of QSR-NVivo 10 software (20, 21). The *a priori* codes were derived from the key questions in the interview guide. The interpretative coding approach helped in fragmenting and re-organizing data to identify themes (21). After assigning codes to transcripts, summaries were prepared from the coded data. The summaries were organized into overarching categories and later assigned themes and sub-themes. The process was continuously discussed with the study investigators (including a senior qualitative researcher) for refinement. Patterns were developed within the themes and concepts, and the original data were reflected continuously upon to ensure that participants' views and perceptions were accurately and adequately presented.

For the data analysis, we relied on an interpretative phenomenological approach (IPA) as it provides a detailed overview of the lived experience of the participants, which is not bound by any pre-existing theoretical preconceptions (22). The themes emerged from the linear process of interpretative analysis of data wherein at the first stage, a free textual analysis was done to annotate the interesting or significant responses of the participants, and later on, emergent themes were captured (23). Once the emergent themes were captured, the qualitative researcher looked for the connection between themes and organized those themes in chronological order, the way they emerged in the transcript, and later on, these themes were organized in analytical or theoretical order to make sense of the connection between themes (23). All participant quotes represented below are anonymized but attributed by age and gender.

The data were interpreted based on the language, metaphors, symbols, repetitions, pauses, and context of the participants and the initial reflexivity of the qualitative researcher (22). During data analysis, the researcher constantly referred to reflexivity notes and a field diary prepared during the interviews to make sense of the language, metaphors, and symbols used by the participants. While preparing the reflexivity notes, the researcher was aware of her age, gender, affiliation with the study site (not a BNCHY staff member), the place of the interviews, and how all these might have shaped the participant interviews and interactions.

Ethics approval was obtained from the following research ethics committees: Faculty of Medicine and Health Sciences, University of Nottingham (UK), CCDC (India), BNCHY (India), and SVYASA (India). The participant information sheet and consent form (available in Hindi, Kannada, and English) were read and shared with the potential participants. Written informed consent was obtained from those interested in participating and for digital recording of interviews.

## Results

Fourteen people were interviewed, the mean age was 51 years (SD = 8.5), and 10 were women. The semi-structured interviews ranged from 20 to 68 min in length, and the average duration was 34 min. Table 1 reports the characteristics of the qualitative study participants and reasons for non-participation in the feasibility RCT. One individual declined to complete the non-participant questionnaire because of the lack of time. Therefore, socio-demographics, diet, and physical activity information and reasons to decline were not available for this respondent. Ten out of the 13 people who completed the non-participant questionnaire were female and married, had not received secondary education, and were unemployed. Over three quarters had a family history of diabetes. Smoking and alcohol levels were low, and most reported eating fruit and vegetables regularly and deep-fried food only occasionally or never. None of them reported doing any vigorous intensity physical activity, but most reported doing moderate intensity physical activities three or more times weekly. All of them reported that lack of time prevented them from participating in the trial, with a third also saying that they already had a healthy lifestyle as a reason for not participating.

**Table 1:** Participants' characteristics and reasons for non-participation in the feasibility RCT.

		***N* = 13**
**SOCIODEMOGRAPHICS**		
Age (years)		40–69
Sex	Female	9
	Male	4
Marital status	Single/separated/divorced/widowed	2
	Married	11
Formal education (years)	≤10	9
	>10	4
Employed	Yes	4
	No	9
Gross monthly household income (₹)		8,000–80,000
Family history of diabetes		10
**DIET**		
High fat/deep-fried food	Regularly (at least 3–4 times/week)	3
	Occasionally	5
	No	5
Fruit and vegetables (portions/day)	< 5	8
	≥5	5
**PHYSICAL ACTIVITY (INTERNATIONAL PHYSICAL ACTIVITY QUESTIONNAIRE-SHORT)**		
Low		1
Moderate		12
High		0
**REASONS TO DECLINE PARTICIPATION IN THE FEASIBILITY RCT**		
Do not have time		13
Cannot do Yoga		2
Not interested in doing Yoga		1
Already maintaining a healthy lifestyle		4
Not interested in research		1
Do not want to be put in a group by chance		1

*Values are range or n*.

*This table is based on 13 out of 14 participants who completed the non-participant questionnaire*.

We categorized the ten analytical themes (see Table 2) that emerged from the interview data, which together represented the reasons for non-participation, into personal, contextual, and study-related barriers. These analytical themes show diversity in participants' perceptions about health, their diets and physical activities, and knowledge about T2DM, Yoga, and YOGA-DP feasibility RCT, which influenced their decisions on study non-participation. Some of the themes were present in every interview, such as the lack of time for self-care, and some were present only in a few interviews, such as the trust in other traditional and alternative therapies, spirituality, and religious belief.

**Table 2:** Themes and sub-themes.

**Themes**	**Sub-themes**	**Number of participants in each theme**
**PERSONAL BARRIERS**		
Lack of time for self-care	Household responsibilities and time management	10 women
	Professional responsibilities and time management	4 women and 2 men
Perceived sufficiency of knowledge to prevent T2DM	Awareness of lifestyle change to prevent T2DM	8 women and 3 men
	Perceived inevitability of T2DM due to the family history of diabetes	2 women and 1 man
	Other misconceptions and lack of information about T2DM	2 women and 3 men
Self-management strategies for health	Self-assessment of health based on symptoms	3 women and 3 men
	Self-restraint and control over health	3 women and 3 men
	Use of glucometer and self-management of health	4 women and 1 man
Trust in other traditional and alternative therapies, spirituality, and religious belief	Faith in other traditional and alternative therapies, such as Ayurveda, meditation, and music	3 women and 2 men
	Reliance on spirituality and religious belief	2 women
**CONTEXTUAL BARRIERS**		
Social influences on health-related decision-making	Male family members had a bearing on women's health-related decision-making	6 women
	Influence of family on health-related decision-making	6 women and 1 man
	Influence of peer group on health-related decision-making	3 women and 2 men
**STUDY-RELATED BARRIERS**		
Lack of information about the study	Unclear understanding of the study	2 women and 1 man
	Lack of information on the study process	5 women and 1 man
	Wrong perceptions about the outcome of the screening test	2 women
Accessibility of the study site	Distance between home and the Yoga center	5 women and 1 man
Lack of trust in the study methods	Lack of trust in the accuracy of the glucometer (used at the time of screening)	2 women and 1 man
Lack of trust in the intervention	No faith in Yoga as a preventive measure for T2DM	2 women
	Fear of Yoga-related adverse events	1 woman

Personal barriers included personal reasons (e.g., household and professional responsibilities, self-management of health, and trust in other traditional and alternative therapies), which acted as barriers to study participation. Contextual barriers were defined as those related to social influences on health-related decision-making, as well as a lack of awareness about routine blood tests and T2DM. Study-related barriers were specific to the design of the study, such as the lack of information about the study, the distance between their residences and the Yoga center, and the lack of trust in the intervention.

## Personal Barriers

### Lack of Time for Self-Care

This theme describes the various household and occupational responsibilities, which made it difficult for participants to participate in the study. Due to the lack of family support and family responsibilities, it was difficult for female participants to spare time for self-care. It was more difficult for women who had additional professional duties, and before and after working hours they had to look after their families. On weekends, they had household tasks which they could not complete on weekdays. Female participants had to take care of their children, husbands, and others in the family, due to which it was difficult for them to spare time for self-care. They also reported that their morning schedules were quite busy as they had to send off their children to school and their husbands to work. They mentioned their inabilities to participate in the study especially in the morning.

“*In the morning, I am so busy that I even do not pick up any call… The usual routine of homemakers is too hectic between 9:30 and 10:00 in the morning, even if they have some problem, they are not able to give time for themselves as husband and children are more important for them*.” (Age: 43; Female)

Many of the female participants mentioned they were involved in household chores, which at times involved moderate intensity physical activities like sweeping and mopping. Thus, they did not feel the need to participate in anything more.

“*I have to take care of my kids. Yes, I do go for a walk and do the household work, which also requires full-body movement. I do the household work from 5 am till 11 pm. I do not take any help from anyone and do everything on my own…Right now, I am unable to find time for this (study)*.” (Age: 42; Female)

One male participant also reported that he traveled long distances to reach the office. He not only had to start early in the morning but also returned quite late in the evening, which is why sparing time to participate in the study would have been difficult for him.

“*My wife gets up early in the morning for the prayers; I sit with her in it. After that, I get my son ready for school and give him a shower. (After morning hours) I have to work from 2:45 to 5:00 pm, and I leave the canteen at 5:30 pm and return home by 6:30 pm. So, it takes 1 hour in the morning and evening to go to the office and come back*.” (Age: 42; Male)

The male participants reported that they got support from the female members (wives and mothers) in the family in their routine tasks. Compared to women, men appeared to have fewer responsibilities at home, however, they were mostly busy with their routine and professional responsibilities, which hindered their participation in the study.

“*I get up at 5:30 am. I get fresh, and I go for a walk for an hour. Upon returning, I follow my daily routine like bathing, having breakfast, and then leave for work. So, this is my routine, and in between when I get time, I practice (singing)*.” (Age: 51; Male)

### Perceived Sufficiency of Knowledge to Prevent T2DM

This theme describes participants' understanding and knowledge of T2DM, which had a bearing on their decisions to decline to participate in the study. Perceived sufficiency of knowledge to prevent T2DM was the most significant factor affecting participants' decisions not to participate in the study. Participants gained knowledge of T2DM from various sources, including family members, friends, social media, and television. They mentioned that they knew enough about T2DM through these sources and thus did not need to participate in the study.

“*Yes, we discuss among ourselves only. We get the information from the talk that we have among ourselves. I do not have much information, but whatever I get to know, it's from there only.”* (Age: 51; Male)

Most of the participants had a clear understanding of the causes of T2DM, which included unhealthy dietary habits, lack of physical activity, heredity, and stress. However, some others had a misconception about dietary practices and blood tests, which influenced their decision-making about health. Misconception and inadequate information prevented some from participating in the study. Some of them also did not have much information about T2DM, which impacted their health-seeking behaviors.

Some participants ignored their family histories of diabetes and did not adopt any specific measure for its prevention. In a few cases, despite having a family history of diabetes, the participants did not have adequate information about the causes of T2DM and precautions to be taken to prevent the condition. Some participants were reluctant to participate in the study due to ignorance about family history of diabetes and lack of information about the prevention of T2DM.

*Interviewer: “Okay. You shared that your mother has diabetes, and she takes medicines as well. Because you live with her, you must have some idea about diabetes. Would you like to tell me something about what you know about diabetes?”*

*Participant: “I have no specific knowledge about it. I just take precautions. I can only do things that are under my control, and I pay attention to what I can do. Otherwise, I do not have much knowledge about diabetes as my mother developed it recently. Also, she doesn't have any problem, though she was diagnosed (with diabetes)*.” (Age: 45; Female)

Quite often, the information participants received from social media and friends was not verified by a clinician. The diversity of information from unreliable sources, such as social media platforms, generated a sense of fear about the blood test. Some participants felt that they would have been diagnosed with T2DM had they gone for a routine blood test; thus, they avoided it. They also followed dietary restrictions based on the information they gathered from their friends and social media without consulting a dietician.

“*People get their routine check-ups done, and they get to know that they are diabetic, but why it happens no one knows. If I tell the truth, I have not gathered any information from anyone about why it happens. No one bothers until they develop the condition (laugh). No one worries about that*.” (Age: 51; Male)

“*I am also having the same kind of information that it happens because of eating more sugar and not working hard, if we take white sugar, it results in an increased sugar level in our body. It has happened to so many people, and they know that it is good to avoid sugar…*” (Age: 67; Male)

Some of the participants also mentioned that they had a family history of diabetes, and they mentioned that diabetes was inevitable as it was there in their families. Thus, they decided not to participate in the study.

“*My father has diabetes. His sugar level was high. It can happen to anyone in the family. Since my father has it, and we are five sisters, so anyone of us can develop diabetes. This is also one of the reasons why we have stopped taking sugar or we take very light sugar*.” (Age: 47; Female)

In some cases, they had the right information, but five of the participants were found to have misconceptions about T2DM. These misconceptions were related to dietary restrictions and the inevitability of T2DM because their family members had diabetes.

### Self-Management Strategies for Health

This theme captures participants' beliefs about their healths and how they dealt with health-related challenges. Many participants assessed their healths based on the sudden development of symptoms. They accordingly took action to manage it and decided to go for medical consultations and tests. Because of their self-belief that they could prevent T2DM like any other condition, many did not feel the need to participate in the study.

“*There is too much heat in my body, and the body stops producing enough blood when April starts. I start feeling weak and dizzy. My veins swell up and blood circulation slows down. I also got an ultrasound done as I was not able to walk*.” (Age: 54; Female)

“*I thought that I should control it myself by quitting sugar completely. I shall be able to control it as it is just the beginning. One can control it*.” (Age: 47; Female)

Some of the participants also decided to not come for the confirmatory venous blood test after their capillary blood tests using a glucometer at the screening camps and take part in the study based on their own assessments of health. They felt that they were doing well health-wise, and thus, they did not need to go for any medical consultation or test or participate in the study.

“*No, I felt that I didn't need the test that much. I personally felt that there is not much problem and by self-control, I could rectify that (control sugar level*).” (Age: 45; Female)

A few participants used a glucometer and blood pressure monitor at home to monitor their blood glucose and blood pressure levels, respectively. These machines gave them a sense of control and power over their healths, and they felt that they were able to take charge of their healths. They trusted these machines for all their health-related problems, and they felt that they could prevent T2DM without participating in the study.

“*I have a BP machine, sugar machine, therapy machine at my home. Everything is there because my husband was sick. I do the blood test myself*.” (Age: 60; Female)

### Trust in Other Traditional and Alternative Therapies, Spirituality, and Religious Belief

This theme describes the participants' trust in other traditional and alternative therapies. Some of them also relied on spirituality, meditation, and music to prevent T2DM. Many participants reported using other traditional and alternative therapies for the prevention of T2DM. They followed the advice of Ayurvedic medical doctors and had Ayurvedic medicines, as prescribed to them. Since they had been using these medicines to prevent T2DM for a long time and perceived the beneficial impact, they decided to continue with these medicines rather than participating in the study for the same.

“*So, the doctor asked me if someone had adviced me to take neem tablets. I told him that a medical consultant at Patanjali (Ayurvedic health and medical center) adviced me to take these pills for 1 month. I had these continuously for 3 months as I was not aware that it would reduce sugar levels below the normal range. Patanjali's consultant later adviced me to stop it, and I did not take it further. (But) it is Ayurvedic; there is no side effect*.” (Age: 42; Male)

A few participants followed their spiritual beliefs and trusted their Gods and spiritual leaders for their healths. They felt that God could make them healthy, and thus, they were reluctant to participate in the study.

“*Everyone suggested me to take care of my health as hepatitis was infectious, but I ignored my health… I had trust in Baba (God) that everything will happen with his wishes*.” (Age: 60; Female)

Similarly, some participants perceived that meditation played an essential role in keeping their bodies active and increasing the alertness of their minds. They even replaced medical consultations, blood tests, and medications with spirituality, meditation, and music. Their reliances and trust in spirituality, meditation, and music made them decide against participating in the study.

“*I have full faith in music. Music is a type of therapy. I will advice doctors as well that medicine is fine, but they should recommend music as it can be helpful in a lot of problems… I never needed to go for the routine check-up… If I ever have headaches or stress, I either sing or listen to music. I cure myself like that*.” (Age: 51; Male)

## Contextual Barriers

### Social Influences on Health-Related Decision-Making

Male participants said they took health-related decisions independently. However, many female participants mentioned that they shared the blood test report from the study screening camps with the male members of the family, including their husbands and sons. These family members appeared to play a significant role in their health-related decision-making as well as their decisions about participation in our study.

“*I did not discuss it with anyone, but only with my husband. He said: if you have a problem then go, but if you do not have any problem and you want to go, then it's your wish. I do not have any problem with that*.” (Age: 40; Female)

Many of them did not perceive the need for a routine blood test in the absence of any disease or symptoms, whereas others got these blood tests done only for the diagnosis of disease when recommended by their physicians and not specifically for T2DM. Thus, not many participants went for a routine test on their own to prevent T2DM. They also mentioned that they got their routine blood tests done when some institutes or hospitals organized free health check-up camps in their residential areas. Most of them viewed the blood test as necessary only to diagnose a disease and not to prevent it, and thus, were not interested in coming for the confirmatory venous blood test.

“*This is quite normal that people do not pay heed to such things (blood test) until they develop some health-related issues… For instance, when I visited the nearby dispensary, they told me that it doesn't happen very often, but at times there is a risk of developing cancer. So, I did not delay it any further and visited the Lady Harding Hospital the same week for all the check-ups. Nobody pays attention until there is a problem (laughed)*.” (Age: 43; Female)

Some participants reported being the office bearers of a Residents Welfare Association (RWA), which helped BNCHY in organizing the screening camps, due to which they were under obligation to undergo the blood test, which otherwise they would have avoided. They did not have any other motivation to visit the study screening camps.

In some cases, participants declined to participate in the study as nobody from their peer groups was interested in going for a venous blood test as part of the study. Their peer groups significantly influenced the decision to not participate.

“*I was alone here, no one else was with me… she (friend) left after the 1st day, and I did not go because I was alone*.” (Age: 44; Female)

## Study-Related Barriers

### Lack of Information About the Study

Some of the participants were neither aware of the study consent process nor did they have a clear understanding of the study. Many of them mentioned that they knew only about the free blood test camps being organized. They were also unaware of the recruitment process and a venous blood test following the capillary blood test. It affected their decisions to participate in the study.

“*No, I did not get that (information sheet). They only asked me to provide my signature.”* (Age: 47; Female)

“*Perhaps, I guess they had given it to me. I do not remember because if I had received it, I would have read it in my free time. In the afternoon, I am always free at home. I do not go anywhere.”* (Age: 67; Male)

Some of the participants said they did not have clear information about their blood test reports at the screening camps. Some of them were also not aware of the difference between being in the high-risk category and having T2DM. One of the female participants also mentioned that she did not want to participate in the study as the screening test showed that her blood glucose level was high. She did not want to come for another test to confirm the increase in blood glucose level as that might have caused stress. Thus, the mistaken perceptions about the outcome of the screening test among participants also prevented them from participating in the study.

“*It is not about participation, but I thought that since it has increased why to go for any further test if that (test) will also show that the sugar level has increased, I may start worrying, so just to avoid that I did not go for your study*.” (Age: 46; Female)

### Accessibility of the Study Site

Many participants mentioned that they decided not to participate in the study due to the long distance between their residences and the health facility. Especially for female participants, it was difficult to leave home and children for 2 h and spend time for self-care. Also, it was difficult for female participants to commit themselves to the study for 6 months. Most of the participants, when asked to suggest changes to the study, requested complete home-based supervised Yoga sessions or Yoga sessions at nearby places.

“*Let me tell you. It is difficult for people from Pandav Nagar to travel there (to the facility). You must know that there is no direct bus service from here to that place*.” (Age: 67; Male)

“*If this program is provided at home, I can do it, but if I have to go somewhere else, then I shall not be able to give time to it. Because if I go out, I always worry about my children as the times are not good*.” (Age: 42; Female)

### Lack of Trust in the Study Methods

Lack of trust in the accuracy of the glucometer used at the screening camps was reported by some participants. They felt that the device used at the camps reported high blood glucose levels, whereas the glucometers used at their homes did not show such high results.

“*I often check my glucose level every 2–3 months. I got it checked before I came for this study, and it was around 116. Usually, it is 107–108. This blood drop machine (glucometer) always shows the sugar on the higher side*.” (Age: 63; Male)

“*She said that she would never go back (to that camp) to get it tested, as the sugar level was higher when the test was done there*.” (Age: 42; Female)

### Lack of Trust in the Intervention

Some showed their distrust in Yoga as a preventive measure for T2DM. They were of the view that Yoga could not help in the long run as the body stops responding to Yoga after a certain period.

“*It affects at the beginning. But I have seen that people who practice Yoga get stable at some point, and after that, there is no benefit of it*.” (Age: 45; Female)

Some of the participants also mentioned that Yoga was not good for their bodies, and they found it challenging to practice a few Yogic poses because of their surgeries, due to which they declined to take part in the study.

“*I do not like Yoga at all. I do not like Asanas, where you have to bend your body. To practice Yoga, your body needs to be flexible, but my body cannot be that flexible… I can tell you now that on rainy days, my stitches hurt. So, if I do much physical activity or Yoga, I will have more problems*.” (Age: 42; Female)

## Discussion

The study identified several personal, contextual, and study-related barriers that influenced participants' decision not to participate in the feasibility RCT. For the majority of them, personal and contextual barriers influenced their decisions of non-participation. It should be noted that many of these barriers are deeply rooted in society and culture and needs to be addressed accordingly.

In this study, personal barriers, such as the lack of time for self-care and perceived sufficiency of knowledge to prevent T2DM had a significant impact on influencing participants' decision not to participate. In India, household responsibilities are still primarily entrusted to the female members of the family (24); thus, it was difficult for them to spare time for self-care. Previous studies on behavior change among women with T2DM have also highlighted that social and family support is an essential requirement for women in bringing any lifestyle change to improve their healths (25, 26). Due to the family history of diabetes and awareness about the impact of T2DM on health, many participants felt that they knew how to prevent T2DM. Though many participants had the correct information about the causes of T2DM, there were some misconceptions about dietary practices and T2DM, which were manifested in their dietary restrictions. Previous studies have also emphasized that the lack of awareness about T2DM affected patients' self-management of T2DM and other attributes (27–29). Some studies have reported a “knowledge vacuum” as an underlying factor for affecting healthcare behavior among diabetic individuals (27, 28). In this study, we found similar challenges, as participants trusted in their capabilities to prevent T2DM. They had trust in Ayurvedic and herbal medicines due to their perceived absence of side effects. Studies conducted among people with chronic conditions, including T2DM, have reported that they perceived traditional and alternative therapies as effective, safe, and convenient to use (30, 31). Similarly, in this study, the participants not only relied on Ayurvedic medicines to prevent T2DM but also believed that spirituality and music could prevent T2DM; thus, they did not feel the need to participate in the study.

Contextual barriers were embedded in the social context of the participants, which negatively influenced their decisions to participate in the study. The influence of friends and family had a bearing on their health-related decision-making which reflects the findings of a previous study that showed that the family had a great deal of influence on patients' health-seeking behavior (32). In the case of females, their husbands and sons influenced the decision to participate in the study.

Many participants mentioned study-related barriers, which prevented them from participating in the study. The lack of adequate awareness and trust in the study process and methods, such as consent and details of the next steps after the screening prevented many from participating. In previous studies, the lack of awareness about clinical research was shown to make it difficult to recruit and retain participants (33) and the lack of information about the study negatively influenced participant recruitment (34). In our study, some of them also showed their distrust in the Yoga intervention. A previous study conducted among adults in the US from racial/ethnic minority and low-income groups also identified that Yoga was perceived as a set of stretching exercises and having no role in weight loss, and these pre-existing beliefs resulted in non-participation in a Yoga program (35). For some participants, perceived difficulty in practicing some Yogic poses prevented them from participating. The distance between the participants' residence and the Yoga center was also a challenge for some participants. These study-related challenges require context-specific strategies. Rapport building and detailed explanation of the study process and methods may help in gaining participants' trust, which could positively impact their decisions to participate in similar studies.

## Strengths and Limitations of the Study

To the best of our knowledge, this was the first qualitative study identifying and exploring the reasons for non-participation in a clinical trial evaluating a Yoga intervention. The qualitative researcher was familiar with the context and extensively engaged with the participants through interviews and observations, which helped in collecting holistic and nuanced data and allowing findings and themes to emerge following data analysis. As described by Smith and Osborn, IPA is idiographic in its examination of the detailed experiences of the individuals (22), and it helped us unravel topics that were “complex and ambiguous and emotional laden” in many instances. It provided an understanding of how participants were making sense of their personal and social worlds and the meanings associated. To ensure the reliability and validity of the research findings, the researcher was constantly involved in a reflexivity exercise about biases, values, assumptions, and their effects on decisions related to different phases of data collection and analysis. A study limitation was the low response rate. Out of 61 people who declined to participate in the feasibility RCT, only 14 agreed to participate in this qualitative research. Most of the respondents in this study were female, which might have influenced the findings. However, within the sample, we managed to interview participants from different socio-demographic backgrounds and achieved data saturation.

## Conclusion

We identified and explored personal, contextual, and study-related barriers to participation in a feasibility RCT in India. The findings will help to address recruitment challenges in future Yoga and other RCTs.

## Data Availability Statement

The raw data supporting the conclusions of this article will be made available by the authors, without undue reservation.

## Ethics Statement

The study was reviewed and approved by the following research ethics committees: Faculty of Medicine and Health Sciences, University of Nottingham, UK; CCDC, India; BNCHY, India; and SVYASA, India. The participants provided their written informed consents to participate in this study.

## Author Contributions

KC conceptualized and designed the study with the help of SG, TH, MH, SL, NK, DH, NT, SK, and DP. PM collected and analyzed the data and wrote the first draft of the manuscript with the help of KC and SG. KC, SG, TH, MH, SL, KS, RN, SM, NK, DH, NT, SK, and DP contributed significantly to the revision of the manuscript. All authors read and approved the final manuscript.

## Conflict of Interest

DH is the Managing Director of a company Harper Public Health Consulting Limited. The remaining authors declare that the research was conducted in the absence of any commercial or financial relationships that could be construed as a potential conflict of interest.

## Publisher's Note

All claims expressed in this article are solely those of the authors and do not necessarily represent those of their affiliated organizations, or those of the publisher, the editors and the reviewers. Any product that may be evaluated in this article, or claim that may be made by its manufacturer, is not guaranteed or endorsed by the publisher.
